# Regulation of gap junction channels by infectious agents and inflammation in the CNS

**DOI:** 10.3389/fncel.2014.00122

**Published:** 2014-05-09

**Authors:** Paul Castellano, Eliseo A. Eugenin

**Affiliations:** ^1^Public Health Research Institute (PHRI), New Jersey Medical School, Rutgers The State University of New JerseyNewark, NJ, USA; ^2^Department of Microbiology and Molecular Genetics, New Jersey Medical School, Rutgers The State University of New JerseyNewark, NJ, USA

**Keywords:** hemichannel, astrocytes, HIV, microglia, oligodendrocytes

## Abstract

Gap junctions (GJs) are conglomerates of intercellular channels that connect the cytoplasm of two or more cells, and facilitate the transfer of ions and small molecules, including second messengers, resulting in metabolic and electrical coordination. In general, loss of gap junctional communication (GJC) has been associated with cellular damage and inflammation resulting in compromise of physiological functions. Recently, it has become evident that GJ channels also play a critical role in the pathogenesis of infectious diseases and associated inflammation. Several pathogens use the transfer of intracellular signals through GJ channels to spread infection and toxic signals that amplify inflammation to neighboring cells. Thus, identification of the mechanisms by which several infectious agents alter GJC could result in new potential therapeutic approaches to reduce inflammation and their pathogenesis.

## Introduction

Gap junction (GJ) channels are formed by connexins (Cxs), a family of proteins with more than 21 members in humans (for review comparing mouse and human Cxs, see Willecke et al., 2002). Each channel is formed by two hemichannels which are hexamers of homologous subunit proteins, termed Cxs (For reviews on structure and function, see Bennett et al., 1991; Saez et al., 2003). Unopposed hemichannels (uHCs) can be formed by one (homomeric connexons) or several (heteromeric) types of Cxs. GJ channels can be formed by two identical, homotypic, or different, heterotypic, subunits of hemichannels. These multiple combinations enable channels formed by different Cxs to vary in their biophysical properties and permeability (reviewed Harris and Bevans, 2001). The large internal diameter of the pore is around 12 A°, and allows ions and intracellular messengers to diffuse between connected cells, including inositol trisphosphate (IP_3_), calcium, cyclic nucleotides, metabolites, toxic molecules, neurotransmitters, viral peptides, and electrical signals (reviewed Saez et al., 2003; Bennett and Zukin, 2004). Through the diffusion of these second messengers, GJs coordinate several physiological functions including electrotonic properties, secretion of glucose, uptake and diffusion of glutamate and other neurotransmitters (reviewed Bennett et al., 1991; Saez et al., 2003). uHCs can also be opened on the cell surface, providing autocrine and paracrine communication systems. Some of the molecules released from the cytoplasm into the extracellular space through the opening of uHC are ATP, prostaglandin E_2_ (PGE_2_), glutamate, aspartate and ions (reviewed Wang et al., 2013; Lohman and Isakson, 2014).

**Figure 1:**
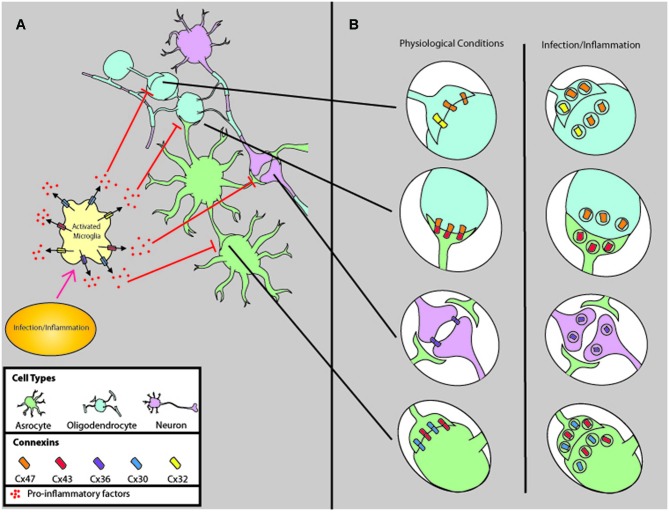
**Comparison of gap junction connectivity under physiological and infectious/inflammatory conditions. (A)** Normally, all cells of the CNS are coupled by GJ to coordinate several physiological functions, including metabolic and electrical coordination. However, upon infection/inflammation GJC is reduced by a mechanism that involves microglia activation and release of pro-inflammatory factors such as PGEs, ATP, neurotransmitters, cytokines, and chemokines. **(B)** Corresponds to the changes in GJ synaptic connections in response to inflammation and infectious agents. Most of these factors induce internalization, degradation, and reduction of GJC.

Within the CNS, Cxs are highly expressed in all cells including brain microvascular endothelial cells, astrocytes, oligodendrocyters, microglia, and neurons (Figure 1). Cx43 and Cx30 are the main Cxs found in astrocytes, while neurons mostly express Cx36, Cx30.2, and Cx45. Oligodendrocytes express Cx29, Cx32, Cx31.3, Cx45, and Cx47, and upon activation microglia express Cx32, Cx36, and Cx43 (For a detailed review of Cx expression and function in CNS parenchyma under normal physiologic conditions and diseased conditions, see Eugenin et al., 2012). Thus, alterations in Cx expression and gap junctional communication (GJC) have a large impact upon CNS function, including response to injury, behavior, synaptic and blood brain barrier (BBB) stability (Eugenin et al., 2012). In this review we will describe how several pathogens dysregulate Cx expression in the CNS.

## Parenchymal GJC is shutdown in response to most infectious agents and associated inflammation

It is accepted that astrocytic GJC contribute to the stability of neuronal networks by favoring metabolic and electrical cooperation between connected cells. However, upon release of classical inflammatory mediators, including IL-1β (John et al., 1999; Duffy et al., 2000), NO (Bolaños and Medina, 1996), ATP (Meme et al., 2004), TGF-β (Reuss et al., 1998, 2000), endothelins (Giaume et al., 1992), and acidification (H^+^ and lactic acid) (Abudara et al., 2001), expression of Cxs and GJC is reduced or totally shutdown. With respect to infectious agents, it is known that *S. aureus* infection or lipopolysaccharide (LPS) treatment of astrocytes reduces Cx43 and Cx36 expression and GJC. Herpes simplex virus-2 (HSV-2) reduces expression of connexins and GJC by direct tyrosine phosphorylation of Cx43 (Crow et al., 1990; Filson et al., 1990; Fischer et al., 2001; Musée et al., 2002; Koster-Patzlaff et al., 2007, 2009; Karpuk et al., 2011). Bovine papillomavirus type 4 E8, when bound to ductin, causes loss of GJC in primary fibroblast (Faccini et al., 1996; Ashrafi et al., 2000). Swine Flu virus down regulates endothelial Cx43 expression by a extracellular-signal-regulated kinase and c-Jun N-terminal kinase dependent mechanism (Hsiao et al., 2010). Borna virus infection of the CNS reduces Cx43 and Cx36 expression (Koster-Patzlaff et al., 2007, 2009). Reduction of Cx36 expression in Huntington’s disease (Petrasch-Parwez et al., 2004) and global reduction of Cx43, Cx32, and Cx47 in Balo’s disease (Masaki et al., 2012) are prominent features in these disorders.

As described above, several disorders and pathogens reduce Cx expression and GJC. In the next sections we will describe how dysregulation of connexin containing channels participate in the pathogenesis of several CNS diseases and the potential role of infectious diseases in the physiological functions mediated by GJC. Our hypothesis is that infectious agents and associated inflammation through GJ impairment can impede upon neuronal myelination, plasticity, migration, and cellular differentiation.

## Cx expression and GJC in parenchymal cells are down regulated by infectious agents and associated inflammation

### Cx expression and GJC are essential for neuronal plasticity

GJ channels contribute to coordinated electrical signaling by serving as a low resistance bridge to transfer electrical signals that influence the receptor components of receptor neurons. The receptor composition of neuronal post-synaptic membranes is highly plastic and influenced by the frequency and strength of signaling from effector neurons. GJs containing Cx36 in hippocampal neurons contribute to neuronal plasticity by participating in synchronizing sub-threshold oscillatory activity essential for long term potentiation (Placantonakis et al., 2006) (for review of electrical activity in Cx KO models see Söhl et al., 2004). Furthermore, close proximity of GJs and NMDA receptors allows for the reciprocal regulation of both synapses (Pereda et al., 2003; Rash et al., 2004). Loss of Cx36 GJC in knockout mice did not involve changes in the slope of excitatory post synaptic potentials (EPSPs) in the hippocampus, but was likely due to increased NR2A/NR2B ratios of NMDA receptor subunits (Wang and Belousov, 2011) indicating that electrical synapses have a major role in regulation of glutamatergic synaptic plasticity in the hippocampus. Borna disease virus infection induces long term reduction of Cx36 mRNA and protein (for up to 8 weeks) in neurons of infected and even in uninfected regions of the hippocampus, which is accompanied by release of pro-inflammatory cytokines that further induce neuronal death (Koster-Patzlaff et al., 2009). LPS also reduces Cx36 expression and GJC in the hippocampus and neocortex (Dobrenis et al., 2005), and Huntington’s disease mouse models had reduced Cx36 expression in the retina (Petrasch-Parwez et al., 2004). Therefore, pathological conditions that reduce Cx36 GJC and expression, such as Borna disease virus (Koster-Patzlaff et al., 2009), Huntington’s disease (Petrasch-Parwez et al., 2004), and inflammation (Dobrenis et al., 2005) have the potential to hinder synaptic plasticity.

As indicated previously, Cx43 and Cx30 are the predominant Cxs expressed in astrocytes, and form GJs that serve critical roles in plasticity, extracellular synaptic metabolite recycling, neuronal excitability, immune activation, inflammation, and BBB integrity (reviewed Eugenin et al., 2012). Electrophysiological recordings of the somatosensory barrel cortex in astrocyte-specific conditional Cx43 KO mice do not show increased amplitude of low frequency potentials (LFP) observed in control mice indicating astrocyte Cx43 is essential for LTP (Han et al., 2014). LPS treatment or *S. aureus* infection reduces GJC in astrocytes for up to 16 h post-infection (Karpuk et al., 2011), followed by reduction in Cx43 expression after 24 h (Esen et al., 2007) indicating that these inflammatory factors have profound effects upon synaptic plasticity and subsequent congnitive functioning.

Abnormal GJC and Cx expression may also influence cognitive function beyond plasticity. Conditional Cx43 KO mice displayed a reduction in environment exploration and increased anxiety based on performance in slit and open-field observations (Han et al., 2014), suggesting that Cx43 dysfunction due to bacterial infection or inflammation may contribute to abnormal cognitive functioning. Moreover, juvenile mice bearing a missense mutation of oligodendrocyte Cx47 also displayed increased anxiety in open field observations, and Cx30/Cx47 double knockouts had severe motor impairments (Han et al., 2014). Therefore, cognitive and motor functions can become impaired during inflammation due to reduced Cx expression and GJC. Our proposal is that infectious agents and subsequent inflammation in the CNS compromises Cx expression and GJC, and repeated bouts of inflammation can have profound effects upon anxiety, learning, and memory.

### Cx expression and GJC are essential for stem cell migration and differentiation

Damage to the CNS from traumatic injury, inflammation, or resection requires the cellular repopulation of neurons to infected areas, but neuronal communication is never fully repaired. Multipotential stem cells have the ability to repopulate damaged parenchyma, but inflammation reduces their ability to migrate and differentiate where needed. The adult CNS has been identified to contain stem cells in the subventricular zone (SVZ), and attempts have been made to utilize the multipotential nature of these cells to repair and repopulate areas of CNS damage (for a review of neurogenesis and migration involving the SVZ, see Brazel et al., 2003). Stem cells of the CNS require Cx expression for differentiation into mature cells (Yang et al., 2005; Hartfield et al., 2011; Lemcke et al., 2013) by allowing glial Cx43 expressing cells to provide a scaffold for directing migration (Miragall et al., 1997; Elias et al., 2007; Cina et al., 2009; Kunze et al., 2009; Marins et al., 2009). In adult mice, neuronal progenitors have been identified as originating from the anterior portion of the SVZ (Doetsch et al., 1997). These progenitors travel through the rostral migratory stream (RMS) and give rise to neurons in the olfactory bulb (Lois and Alvarez-Buylla, 1994). A study attempting to identify proliferating cells of the RMS and olfactory bulb identified that BrdU positive cells correlated with lower levels of Cx43 expression in adult mice (Miragall et al., 1997). However, it cannot be assumed that the BrdU positive cells are actively replicating, and may have possibly already received the signaling needed for differentiation into their final cell fate. This correlated with a study in which knockdown of Cx43 in a neural differentiation culture model had significantly reduced proliferation and differentiation (Lemcke et al., 2013), and neurospheres obtained from Cx36 knockdown had reduced numbers of differentiated neurons and coupling (Hartfield et al., 2011).

Interestingly, Cx43 appeared to form a scaffold in the RMS/Cx43/Brdu^+^ study (Miragall et al., 1997), which may correlate with the dependence of Cx43 for migration. Repopulation of hippocampal neurons and glia occurs from the migration and differentiation of radial glia (RG)-like precursors that are dependent upon Cx43 and Cx30, as shown through conditional KO and viral ablation models in which there were reductions in Prox1^+^ cells, NeuN and Ki67 staining in the dentate gyrus (Kunze et al., 2009). Therefore, repopulation of hippocampal parenchyma depends upon Cx43 and Cx30 expression, suggesting that chronic inflammation or pathogenesis in this region could impede migration and repopulation after injury or inflammation by a Cx dependent mechanism.

Cx43 is essential for neuronal migration in the developing brain, and fetal infection/inflammation that reduces Cx43 expression can have devastating consequences. Using a conditional KO model in which the c-terminal tail of Cx43 was deleted in nestin^+^ cells demonstrated that neurons did not migrate to the cortical plate, and halted at the intermediate zone (Cina et al., 2009). Proper migration depends on surface expression of Cx, but not GJC, as seen using a mutant model in which the conserved tyrosine in the third transmembrane domain of Cx43 and Cx26 was deleted. This deletion prevents the opening of GJ channels, but Cx participation in surface adhesion and migration is conserved (Elias et al., 2007). Therefore neuronal migration depends on both surface expression and an intact c-terminal tail of Cx43, but not GJC. Reduced expression of Cx43 during inflammation of the neonatal brain could have permanent effects from impaired neuronal migration, such as ganglion cell layer deterioration observed in congenital ocular toxoplasmosis from *T. gondii* infection (Safar et al., 1995), and reduced white matter area and atrophy seen in adult brains after fetal influenza infection (Fatemi et al., 2008). We propose that reducing inflammation from infectious agents or maintaining Cx expression within the CNS will increase progenitor cell migration and neuronal differentiation by maintaining the Cx scaffold needed for migration and also by allowing differentiation signals to correctly influence progenitors.

### GJC is essential for proper myelination

Myelin is essential for rapid neuronal signaling by contributing to saltatory conduction. Without myelination of axons, depolarization dissipates in a distance dependent manner and signals cannot be conveyed to post-synaptic membranes. Loss of myelination is a hallmark of various diseases that result in motor and cognitive deficit, such as multiple sclerosis (MS), amyotropic lateral sclerosis, and Alzheimer’s disease, but the contribution of infectious agents upon demyelination has not been examined.

Oligodendrocytes are responsible for myelinating neurons in the CNS, and astrocytes provide metabolic support of this process. Homotypic Cx47 and Cx32 GJs couple oligodendrocytes to themselves, and heteromeric Cx47/Cx43 GJs couple oligodendrocytes to astrocytes (Figure 1; Rash et al., 2001). Double KO models of Cx47/Cx32 or Cx47/Cx43 have severe myelin abnormalities and die within 90 days (Tress et al., 2012; May et al., 2013), but single knockouts of Cx32 or Cx47 do not result in premature death or alterations in GJC (Menichella et al., 2003). This indicates there may be compensatory function when one Cx is disrupted, and both forms of Cxs need to be down regulated in order for a pathogenic phenotype to arise. In line with the KO model, Experimental Autoimmune Encephalitis (EAE) mouse models induced by intraperitoneal injection with recombinant myelin oligodendrocyte glycoprotein (MOG) had reduced levels of Cx32 and Cx47 during relapsing stages of inflammation (Eugenin et al., 2012; Markoullis et al., 2012). Further evidence using an inducible double KO model indicates lack of astrocytic Cx43 and Cx30 produces widespread myelinic edema and vacuolization, accompanied by hippocampal CA1 region-specific pathology (Lutz et al., 2009). Therefore, localized acute neuroinflammation may have long term effects upon myelination by downregulating Cxs essential for maintaining oligodendrocyte integrity.

Acute inflammation can also induce demyelination through the activation of microglia that secrete toxic mediators. Microglia are resident phagocytic cells of the CNS that migrate to points of infection and injury to engulf debris, clear infection, and amplify inflammation, but they are not always beneficial in eliminating neuropathogenesis without inducing severe damage to myelin. LPS activated microglia reduce the production of myelin basic protein (MBP), and induce oligodendrocyte progenitor cell (OPC) death by releasing TNFα (Pang et al., 2010) through a mechanism mediated by glutamate that opens uHCs (Takeuchi et al., 2006). *In vivo* activation of oligodendrocyte TNFα receptor induces severe myelin vacuolization and death (Akassoglou et al., 1998). Interestingly, in the above study, OPC death did not occur until after 16 h of LPS treatment, *in vitro*. However, after 48 h nearly all OPCs died (Pang et al., 2010), indicating acute inflammation has the capability of reducing remyelination with devastating consequences long after initial infection/inflammation if microglia are not deactivated. Therefore, TNFα induces the release of glutamate through uHCs in microglia (Takeuchi et al., 2006) during chronic or relapsing/remitting inflammation (Figure 1) in the CNS will result in demyelination.

Aside from inducing OPC death, microglial release of TNFα (Pang et al., 2010) may indirectly induce demyelination by dysregulation of GJC (Karpuk et al., 2011) and water content in astrocytes (Sharma et al., 2010). Direct LPS injection into mice spinal cords induces continued demyelination over 30 days with significant down regulation of astrocytic Cx30, Cx43, and Aquaporin-4 (AQP4) after 3 days (Sharma et al., 2010). In post-mortem brain slices of patients with Balo’s disease, a demyelinating condition similar to MS, immunostaining revealed reductions in Cx43, Cx32, Cx37, and AQP4 in concentric lesions, but no reactivity of anti-Cx or anti-AQP4 antibodies were detected (Masaki et al., 2012), suggesting that down regulation of these membrane proteins was likely to contribute to the neuro-pathogenesis of disease but may not be the cause. AQP4 is closely associated with astrocyte GJs (Sharma et al., 2010), and its expression is necessary for proper water balance in the CNS (Alexander et al., 2008; Rama Rao et al., 2014; Wu et al., 2014). Therefore, demyelinating diseases result in down regulation of Cxs and AQP4 that are essential for maintaining myelin integrity.

Reduced expression of AQP4 induces astrocyte swelling (Alexander et al., 2008) and retraction of endfeet (Alvestad et al., 2013) associated with increased brain edema in rat models of hepatic encephalopathy (Jayakumar et al., 2014), which correlated with depolymerization of actin cytoskeleton in cultured human astrocytes (Nicchia et al., 2005). In agreement, whole cell patch clamp of *S. aureus* infected astrocytes have increased membrane capacitance (Karpuk et al., 2012). Therefore, localized acute neuroinflammation may have long term effects upon myelination by downregulating Cxs essential for maintaining oligodendrocyte integrity. In contrast to Balo’s disease, brain slices from human MS patients had increased expression of Cx43 likely due to astrogliosis, but similar reduced expression of Cx32 in chronic active lesions (Eugenin et al., 2012). Therefore, decreased Cx32 expression due to inflammation may be sufficient to impair myelination in humans, and the pathology of MS differs from Balo’s disease.

We propose Cx expression and GJC between oligodendrocytes are essential for myelination, and reducing inflammation rapidly may decrease damage to myelin. As stated above, reduced inflammation will also help reduce the pathogenic effects upon plasticity, progenitor cell migration, and cellular differentiation.

## Cx expression and GJC are enhanced in immune cells to resolve pathogenesis in the CNS

### Activated microglia amplify inflammation and adversely affect neurons by releasing toxic mediators through uHCs

In all CNS diseases, immune cells play a critical role in controlling inflammation. However, unresolved inflammation in the CNS can have devastating consequences, including impaired myelination (Akassoglou et al., 1998; Markoullis et al., 2012) sensorimotor deficits (Han et al., 2014), and loss of signaling that contributes to learning and memory (Koster-Patzlaff et al., 2009; Wang and Belousov, 2011). Activated microglia release pro-inflammatory factors, such as ATP (Orellana et al., 2013) and glutamate (Takeuchi et al., 2006), through uHCs that serve to amplify inflammation with devastating effects if left unchecked. Neuronal beading, a sign of neurotoxicity, was induced by activated microglial release of glutamate from Cx32 uHCs (Takeuchi et al., 2005, 2006). Treatment of cultured neurons with either IL-1β or TNFα reduced the number of neuronal processes and expression of microtubule-associated protein 2. There was also an increase of intracellular and extracellular glutamate production and mitochondrial release of glutaminase, which collectively induced neuronal death after 3 days (Ye et al., 2013). TNFα mediated reduction of excitatory amino acid transporter (EAAT) and glutamate toxicity is also attributed to neuronal death in Japanese encephalitis virus (JEV; Chen et al., 2012), West Nile virus (WNV; Blakely et al., 2009), and Sindbis virus (Carmen et al., 2009). Therefore, release of pro-inflammatory factors through uHCs from activated microglia can have devastating affects upon CNS parenchyma.

### Peripheral immune invasion is necessary for clearance of inflammation in the CNS, and is highly dependent on Cx expression and GJC

Immune responses in the CNS can be broken down into three general phases: first, the secretion of chemotactic factors from damaged areas; second, the migration of immune cells to the point of injury, inflammation, or pathogen invasion; and third, the clearance of debris, infectious agents and recovery. Cx expression and GJC are critical for each of these phases and for generating an adaptive immune response. In monocytes, inflammatory signals induce Cx43 expression allowing migration across the BBB (Eugenin et al., 2003). This is a key step that allows population of the CNS with phagocytic antigen presenting cells that are essential for reducing inflammation (Akassoglou et al., 1998).

Macrophages and dendritic cells (DCs) play an essential role in generating and adaptive immune response for clearing bacterial and fungal infections through phagocytosis, antigen presentation, and release of factors that reduce inflammation. Cx43 is essential for phagocytic activity in peritoneal macrophages as seen in a mouse model implementing immunostaining and confocal microscopy of Cx43, LAMP-2, and dyna beads (Anand et al., 2008), and paralleled phagocytic mechanisms involving Cx43 in monocyte-derived macrophages in the CNS are likely. Phagocytosis serves to clear debris from damaged parenchyma, followed by antigen processing and presentation. A study using CX3CR1^GFP+^ mice (to endogenously label antigen presenting cells) immunized with MOG (to induce neuroinflammation), found CD11c^+^ DCs abundantly infiltrated CNS parenchyma, were positive for myelin antigen after 21 days, and participated in cross-presentation with T-cells (Sosa et al., 2013). Antigen cross-presentation at immunological synapses is critical for adaptive immune response, and membrane bound Cx43 is a necessary component of supramolecular activation clusters (Mendoza-Naranjo et al., 2011). Coupled cells are able to share antigens and trigger response in cytotoxic T lymphocytes even when some cells were never exposed directly to a pathogen (Neijssen et al., 2005). Using a mimetic peptide targeting the extracellular portion of Cx43, DC to T-cell (Elgueta et al., 2009) and DC to DC (Sosa et al., 2013) cross presentation and activation is inhibited *in vitro*, indicating Cx43 is necessary for efficient adaptive immune response in the CNS.

Collectively, these data suggest that immune cells utilize GJs to enhance adaptive immune responses and clearance of inflammation from infectious agents in the CNS.

## Several pathogens use GJC to spread infection and inflammation

In general, parenchymal GJC is shut down in the CNS upon initial inflammation (Koster-Patzlaff et al., 2007, 2009; Karpuk et al., 2011) and followed by reduction in Cx expression (Esen et al., 2007). In a mouse model of *S. aureus* infection, astrocyte GJC was dramatically reduced near the margins of abcesses after 3 days of infection, but there were no significant changes in protein expression of Cx43 or Cx30. At this same timepoint opening of uHC was evident as compared with uninfected contralateral brain slices (Karpuk et al., 2012), indicating that early infection reduces GJC and opens uHCs in astrocytes. Interestingly, the timecourse for uHC channel opening in astrocytes was similar to the timecourse of uHC channel opening induced by amyloid-β treatment. Neurotoxicity induced by amyloid-β was associated with uHC opening (Panx1 and Cx43) and subsequent release of glutamate and ATP (Orellana et al., 2011).

Release of toxic factors through uHCs during infection promotes inflammation and induces neurotoxicity. *In vivo* ATP imaging of rat spinal cord after laminectomy and weight drop injury found a dramatic reduction in the amount of ATP released around the site of injury in Cx43 KO or knockdown models (Huang et al., 2012; O’Carroll et al., 2013), with correlative improvement in Bousso mouse scale (BMS) score for locomotion. However, complete recovery in action potential propagation was not achieved in Cx43 KO mice after 7 days (Huang et al., 2012), which indicates astrocytic Cx43 uHC release of ATP during inflammation is not the only contributor to neuronal damage. Moreover, the release of ATP acts as a chemoattractant for activated, pro-inflammatory microglia that further amplify injury through increased release of toxic factors (for a review of microglia chemoattraction see Chekeni and Ravichandran, 2011). Therefore, infectious agents amplify inflammation from the release of toxic mediators through uHC that attract microglia and amplify inflammation. Thus, we propose that the use of uHC blockers during CNS inflammation will reduce neuronal damage and improve prognosis.

## Participation of GJ and uHC in bystander killing by several pathogens

Bystander killing is the process by which an infected cell induces the death of a neighboring uninfected cell. Bystander killing occurs between microglia-microglia (Ribot et al., 2007), astrocytes-astrocytes (Eugenin and Berman, 2007), astrocytes-neurons (Loov et al., 2012), and astrocytes-endothelial cells (Eugenin et al., 2011), and evidence suggests this process is GJ mediated (Eugenin and Berman, 2007; Eugenin et al., 2011). However, the exact mechanisms involving bystander cell death observed in pathogenic conditions of the CNS are not completely understood.

GJs amplify injury and induce bystander cell death during metabolic stress. Bcl2 is an anti-apoptotic mitochondrial protein, and can provide resistance to cell death when transfected into a glioma cell line. Using a Cx43 double-transfectant (Bcl-2^+^Cx43^+^) and selecting for varying degrees of expression of each protein revealed that bystander cell death due to metabolic stress was in direct proportion to the number and density of GJs with less resistant neighbors (Lin et al., 1998). Therefore, the ability to resist apoptosis from high expression of Bcl-2 did not protect cells from death if a weaker neighbor was compromised, as long as there was active GJ coupling. This was repeated with variations of stress in mono-cultures and mixed cultures, and the same conclusion was drawn (Lin et al., 1998).

Interestingly, HIV may have the ability to control GJC for its own benefit by developing resistance to cell death in cultured human astrocytes, while inducing the death of uninfected bystanders in a GJ dependent manner (Eugenin and Berman, 2013). Bystander cell death was reduced using GJ blockers, as did blocking IP_3_ or cytochrome c (CytC) mediated signaling. CytC is a pro-apoptotic signaling molecule that mediates caspase activation by participating in apoptosome formation, and microinjection of CytC in cultured astrocytes induces 100% apoptosis. However, microinjection of CytC into HIV infected astrocytes did not induce cell death, suggesting that HIV infection protects astrocytes from apotopsis (Eugenin and Berman, 2013). Therefore, HIV infected astrocytes are protected from cell death while killing uninfected neighbors by a GJ dependent mechanism. This is consistent with the low level of chronic inflammation seen in patients with HIV associated neurocognitive disorder (HAND), and indicates HIV may have the ability to control GJs for its own benefit in cell death resistance and dissemination.

The HIV protective effect on astrocytes may parallel cell death resistance in gliomas by a common mechanism involving CytC and Bcl-2. CytC is a key component of the mitochondrial electron transport chain (ETC), and donates an electron to complex IV for the final step in H^+^ transport into the intermembrane space. An early step of apoptosis induced by metabolic stress is the release of CytC from the inner mitochondrial membrane into the cytoplasm, where it interacts with Apaf-1 and activates apoptosome formation. The exact components that selectively induce release of mitochondrial components are currently under investigation, but a series of outer mitochondrial membrane (OMM) proteins have been identified as capable of allowing the release of CytC. Bcl-2 associated X (Bax) protein is a pro-apoptotic cytosolic member of the Bcl-2 family that translocates to the OMM and is associated with CytC release and apoptosis (for reviews of CytC induced apoptosis and the relation with OMM proteins, see Kilbride and Prehn, 2013; Renault et al., 2013). However, in drug resistant gliomas Bax does not translocate, and overexpression of Bcl-2 conveys resistance to staurosporine induced apoptosis (Murphy et al., 2000). Interestingly, resistance to Temozolomide (TMZ), an anti-tumor pharmaceutical studied for treating gliomas, is influenced by Cx43 expression and correlates with mitochondrial alterations including release of CytC and increased Bcl-2/Bax ratios. Knockdown of Cx43 using shRNA in a LN229 glioma cell line increases Bax expression, reduces co-localization of CytC with mitochondria, and conveys reduced TMZ resistance which increases tumor susceptibility to pharmaceutical treatment *in vitro* (Murphy et al., 2000, 2012). Therefore, there is a mechanism by which Cx43 influences mitochondrial signaling pathways involving apoptosis, and HIV may support Cx43 expression and gap junctional intercellular communication (GJIC) to convey a protective effect.

Use of the retroviral beta-galactosidase at gag (BAG) vector and Herpes Simplex Virus Thymidine Kinase (HSV-tk) has expanded our knowledge of the relationship between GJIC and bystander cell death. Several studies validate the use of this retroviral vector in drug delivery intending to preserve brain tissue while treating gliomas, transfected a retroviral BAG vector into rat brain inoculated with a C6 glioma cell line (Short et al., 1990). Vector insertion into glioma cells was much greater than normal brain tissue because only a low level of retroviral incorporation into host DNA is possible in non-replicating cells, while glioma cells are extensively labeled since they rapidly divide (Miller et al., 1990). HSV-tk transfection combined with glanciclovir (GCV) treatment, an antiretroviral pharmaceutical, reduced medullablastoma xenograft tumor volume (Rosolen et al., 1998), but complete eradication was not shown. However, we propose this technique can be used to hunt tumor cells in the CNS for drug delivery after resection.

Combining HSV-tk therapy with induced expression of Cx43 could have promise as a treatment for malignant gliomas after resection. Resection is not always successful in eradicating gliomas because glial tumor cells are able to disseminate and intersperse themselves in brain parenchyma far from the original tumor mass, making resection ineffective in reducing reemergence. Neural stem cells (NSCs) engineered to secrete IL-12, and bone marrow derived stem cells (BMSCs), have shown the ability to track migrating tumor cells (Ehtesham et al., 2002; Nakamizo et al., 2005), thereby allowing the targeting of interspersed tumor cells. Glioma stem cell tumorspheres have low GJC, and inducing the expression of Cx43 inhibited self-renewal and invasiveness (Yu et al., 2011). Moreover, bystander cell apoptosis is increased in proportion to GJ connectivity (Lin et al., 1998). Therefore, combining HSV-tk therapy with induced expression of Cx43 should be a more effective eliminator of undetectable malignant gliomas after resection. Using a rat model in which C6 glioma cells transfected to express Cx43 were injected into the caudate nucleus, followed by injection of BMSC-tk cells into the tumor site and GCV intraperitoneal injection, animals were able to live six times longer and reduced tumor volume by a quarter of the original size was observed (Huang et al., 2010). Therefore, bystander killing of tumor cells induced by the expression of Cx43 shows promise in combination with current therapies.

## Conclusions

We have outlined the involvement of Cx expression and GJC in neuronal plasticity, myelination, migration, stem cell differentiation, and discussed the devastating effects inflammation and infectious diseases can incur on these processes. Inflammation induced by disease is amplified by the opening of uHCs in microglia, and is followed by peripheral immune invasion into the CNS to resolve the damage. Adaptive immune responses following inflammation in the CNS are also dependent on Cx expression and GJC, and the mechanisms of adaptive immune response beyond antigen cross-presentation are an active field of study. In contrast, GJs are also used by some pathogens to induce bystander killing, and this phenomena has been explored to treat malignant gliomas in a novel HSV-tk/Cx43 “hunter-killer” tactic after resection. Thus, further studies to control GJC and uHCs can improve the detrimental effects of infectious diseases and associated inflammation in the CNS.

## Conflict of interest statement

The authors declare that the research was conducted in the absence of any commercial or financial relationships that could be construed as a potential conflict of interest.
